# Supplementary material for: Cyclic oligoadenylate signalling mediates Mycobacterium tuberculosis CRISPR defence
Source: Nucleic Acids Res. 2019 Aug 8;47(17):9259–70. doi: 10.1093/nar/gkz676 (PMC6755085; doi:10.1093/nar/gkz676)
Supplement: gkz676_Supplemental_File [file gkz676_supplemental_file.pdf]

Cyclic oligoadenylate signalling mediates *Mycobacterium tuberculosis* CRISPR defence

Supplementary Information – Grüschow et al.

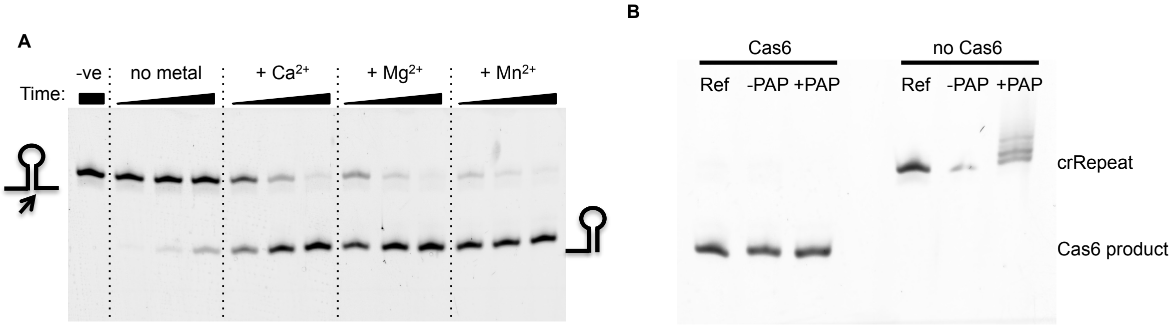

Figure S1 The Cas6 ribonuclease cleaves the *Mtb* CRISPR repeat sequence to generate crRNA. (**A**) Cas6 (0.5 µM) was incubated with 50 nM 5'-FAM CRISPR repeat RNA at 37 °C for 5, 15, 45 min in 20 mM Tris, 100 mM potassium glutamate, pH 7.5 in the absence or presence of 5 mM divalent metal ions as indicated. Reactions were stopped by phenol-chloroform extraction. (**B**) Cas6 cleavage leaves a 3'-(cyclic) phosphate group. CRISPR repeat RNA (crRepeat, 5'-FAM labeled, 400 nM) was digested with 2 µM Cas6 for 1 h in the presence of Mg^{2+} using the same reaction conditions as before. Phenol-chloroform followed by chloroform extraction provided the substrate for the *E. coli* Poly(A) polymerase (PAP, New England Biolabs) reaction. Polyadenylation was performed according to the manufacturer's instructions. In a parallel experiment, Cas6 was omitted. The CRISPR repeat RNA but not the Cas6 product can be 3'-polyadenylated by PAP. This suggests that the reaction product has a cyclic 2',3'-phosphate, as observed for other Cas6 enzymes. This observation, together with the observation that calcium supports enhanced cleavage of the CRISPR repeat, suggests that the metal ion does not participate directly in catalysis but rather plays a role in stabilisation of the RNA substrate or RNA:protein complex. For these experiments, lanes labelled "Ref" show RNA before polyadenylation while -PAP and +PAP show RNA incubated in polyadenylation buffer in the absence and presence of Poly(A) polymerase, respectively.

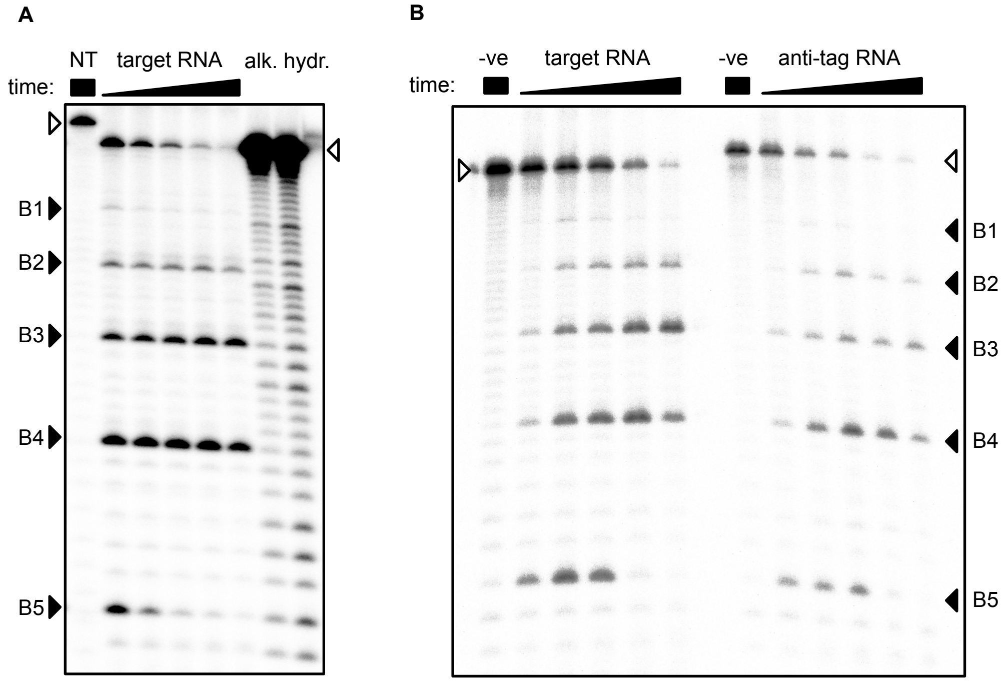

Figure S2. RNA backbone cleavage by Csm interference complex.

(**A**) 5'-^{32}P-labeled target RNA or non-target (NT) RNA were treated with 0.8 µM Csm effector complex for 5, 15, 30, 60, 120 min. Reactions were analysed by denaturing PAGE alongside alkaline hydrolysis ladders prepared from target RNA. (**B**) Target RNA and anti-tag RNA (full-length complementarity to crRNA including repeat-derived 5'-handle) are both cleaved by Csm to give identical products. Reaction time: 0.5, 2, 5, 30, 100 min; substrate RNA is indicated by unfilled triangles; the five cleavage sites (B1 – B5) with the characteristic 6 nt spacing are indicated by filled triangles. Target RNA with a 4 nt truncation at the 3'-end was used, leading to a different distribution of cleavage products compared to Figure 2. It is not clear why product B5 is depleted over time in these gels but not in figure 2, and this phenomenon could be followed up in future studies.

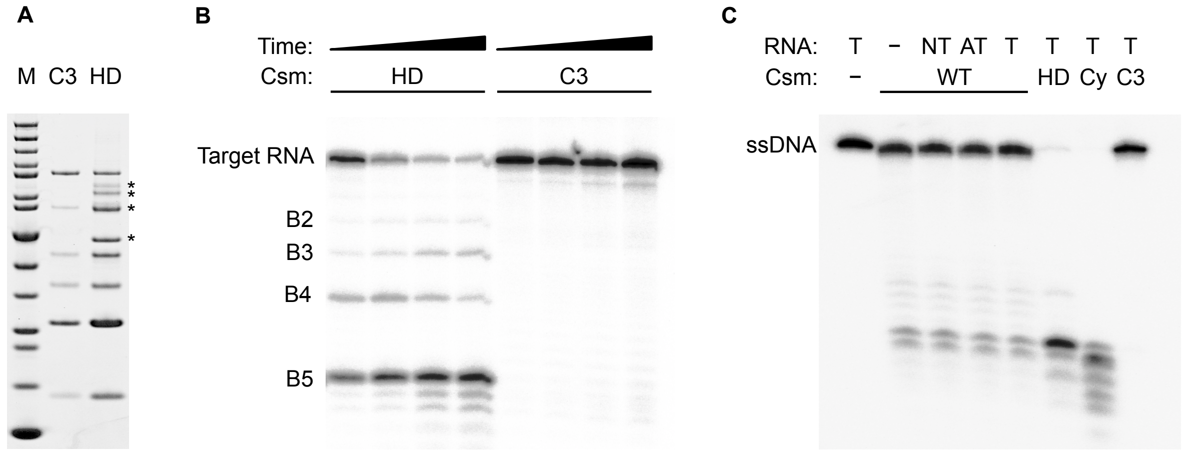

Figure S3. In vitro analysis of Csm HD mutant. (**A**) SDS-PAGE of purified Csm3 D35A (C3) and Cas10 H18A/D19A (HD) variant interference complexes; *: contaminants; M: PageRuler Unstained (Fisher Scientific). (**B**) Target RNA backbone cleavage by Csm3 D35A (C3) and Cas10 H18A/D19A (HD) variant interference complexes; 5'-radiolabeled target RNA was incubated with HD or C3 in the presence of Mg^{2+} for 5, 10, 30, 60 min at 30 °C; as expected the characteristic cleavage products B2 – B5 are produced by HD but not the C3 variant. (**C**) The ssDNase activity of Csm interference complex is not dependent on RNA substrate or active site mutations; 5'-radiolabeled ssDNA was incubated with Csm wild type (WT), HD, Cas10 D630A/D631A (Cy), or Csm3 D35A (C3) in the presence of Mg^{2+} for 90 min at 30 °C; cold RNA was added as indicated. Mn^{2+} and Co^{2+} also supported the observed activity, Zn^{2+} less so, and Cu^{2+} did not stimulate DNase activity (data not shown). T: target RNA, NT: non-target RNA, AT: anti-tag RNA.

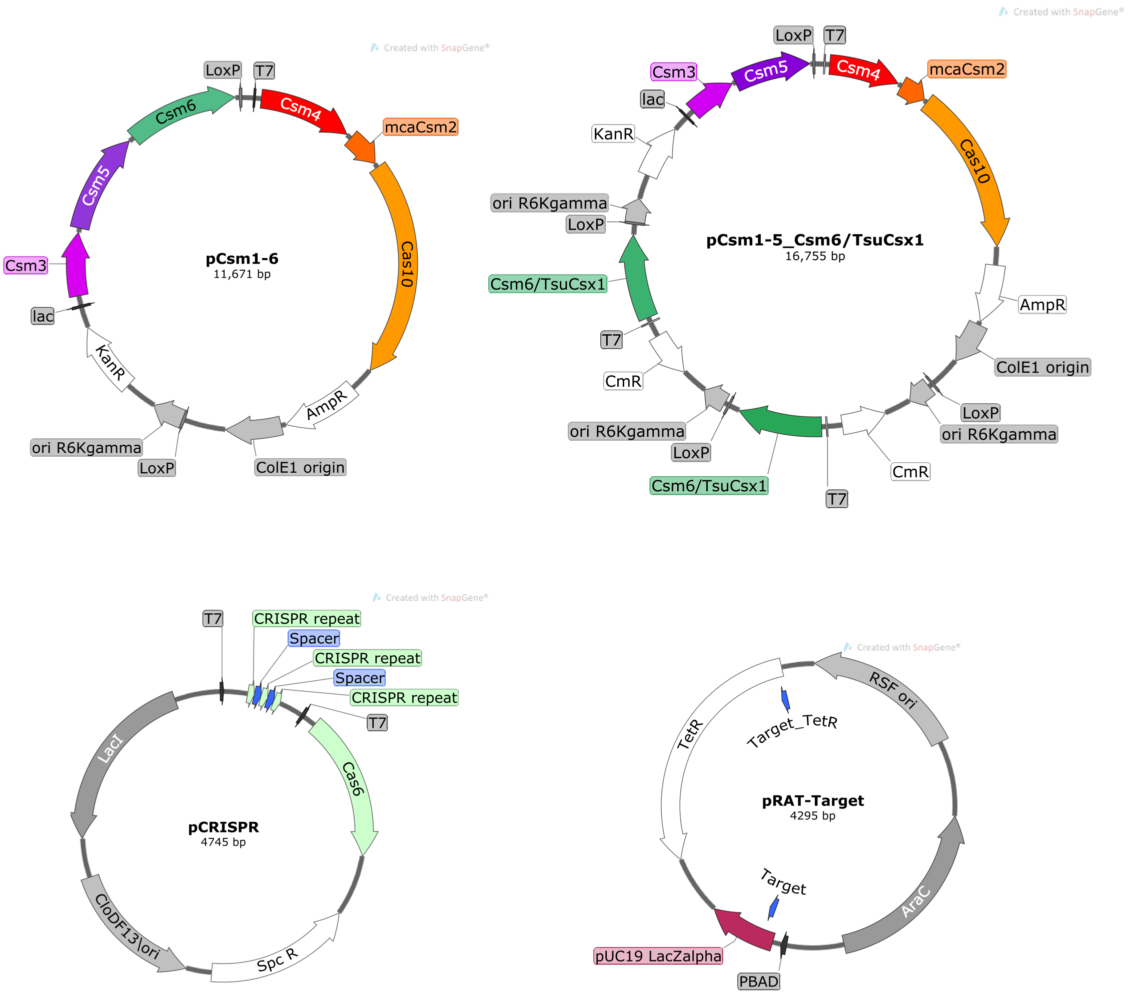

Figure S4. Selected plasmid maps for constructs used in plasmid immunity assays. In pRAT-Target, the blue arrows indicate the positions of the match to crRNA targeting the pUC19 LacZα MCS (Target) or the tetracycline resistance gene (Target_TetR).

Table S1: Plasmids constructed in this study

Name	Genes	Resistance	Notes	Use
pCsm1-5	*csm1, csm2, csm3, csm4, csm5, Mca_csm2*	Amp, Kan, Cm	MultiColi™ pACE, pDK, pDC recombination; ColE1 ori; pT7 (*csm1, 2, 4* on pACE), pLac (*csm3, 5* on pDK), pT7 (Mca_*csm2* on pDC), TEV-cleavable N-His tag on Csm4	Protein production and purification
pCsm1-5_C3	*csm1, csm3 D35A, csm4, csm5, Mca_csm2*	Amp, Kan	MultiColi™ pACE and pDK recombination; ColE1 ori; pT7 (*csm1, 4, Mca_csm2* on pACE), pLac (*csm3, 5* on pDK), TEV-cleavable N-His tag on Csm4	Protein production and purification
pCsm1-5_Cy	*csm1 D630A D631A, csm3, csm4, csm5, Mca_csm2*	Amp, Kan	as pCsm1-5_C3	Protein production and purification
pCsm1-6	*csm1, csm3, csm4, csm5, csm6, Mca_csm2*	Amp, Kan	pLac (*csm3, 5, 6*), otherwise as pCsm1-5_C3	Plasmid immunity assay
pCsm_Control	-	Amp, Kan	pACE and pDK recombination	Plasmid immunity assay; vector control
pCsm1-5_ ∆Csm6	*csm1, csm3, csm4, csm5, Mca_csm2*	Amp, Kan	as pCsm1-5_C3	Plasmid immunity assay
pCsm1-6_C3	*csm1, csm3 D35A, csm4, csm5, csm6, Mca_csm2*	Amp, Kan	as pCsm1-6	Plasmid immunity assay
pCsm1-6_Cy	*csm1 D630A D631A, csm3, csm4, csm5, csm6, Mca_csm2*	Amp, Kan	as pCsm1-6	Plasmid immunity assay
pCsm1-6_HD	*csm1 H18A D19A, csm3, csm4, csm5, csm6, Mca_csm2*	Amp, Kan	as pCsm1-6	Plasmid immunity assay
pCsm1-5_Csm6	*csm1, csm3, csm4, csm5, csm6, Mca_csm2*	Amp, Kan, Cm	pACE, pDK, pDC recombination; pT7 (*csm6* on pDC), otherwise as pCsm1-5_C3	Plasmid immunity assay
pCsm1-	*csm1, csm3, csm4,*	Amp, Kan,	as pCsm1-5_Csm6 but	Plasmid immunity

5_tsuCsx1	*csm5, Mca_csm2, Tsu_csx1*	Cm	*Tsu_csx1* instead of *csm6*	assay
pCRISPR	*cas6,* pUC MCS-targeting CRISPR array (3 repeats, 2 spacers)	Spc	pCDF-Duet-1 derivative; CRISPR in MCS-1, *cas6* in MCS-2	crRNA biogenesis, *in vitro* and *in vivo*
pCRISPR_TetR	*cas6,* TetR-targeting CRISPR array (5 repeats, 4 spacers)	Spc	as pCRISPR	crRNA biogenesis, *in vivo*
pRAT	-	Tet	RSF ori; pBAD	Plasmid immunity assay; control plasmid for pUC MCS-targeting Csm, target plasmid for TetR-targeting Csm
pRAT-Target	pUC19 *lacZ*α	Tet	as pRAT	Plasmid immunity assay; target plasmid for pUC MCS-targeting Csm
pCas6-CHis	*cas6*	Kan	pEHisTEV-derivative; C-terminal His-tag	Protein production and purification
pEHisTEV-Csm6	*csm6*	Kan	pEHisTEV-derivative; TEV-cleavable N-His tag	Protein production and purification
pEHisTEV-TsuCsx1	*Tsu_csx1*	Kan	pEHisTEV-derivative; TEV-cleavable N-His tag	Protein production and purification

Table S2 Oligonucleotides and primers used in this study

Name	Sequence 5' → 3'	Notes
mtbCRISPR array	ggattcggatcctctagagtcgGTCGTCAGACCCAAAACCCCGAGAGGGGACGGAAACGaattcgagctcggtacccggggatccctagg	dsDNA
mtbCA_Rep-5'_t	catggaatagCACGCTGGACGAATTGTCCATAGA	5'-P_i
mtbCA_Rep-5'_b	CTCGGGGTTTTGGGTCTGACGACTCTATGGACAATTCGTCCAGCGTGctattc	5'-P_i
mtbCA_Rep-3'_t	GTCGTCAGACCCAAAACCCCGAGAGGGGACGGAAACgactctaccg	5'-P_i
mtbCA_Rep-3'_b	tcgacggtagagtcGTTTCCGTCCCCT	5'-P_i
mtbCA_TetR-Sp_t	GTCGTCAGACCCAAAACCCCGAGAGGGGACGGAAACtgacggtgccgaggatgacgatgagcgcattgttaga	5'-P_i
mtbCA_TetR-Sp_b	CTCGGGGTTTTGGGTCTGACGACtctaacaatgcgctcatcgtcatcctcggcaccgtcaGTTTCCGTCCCCT	5'-P_i
NdeInsFor	gtttaactttaagaaggagatatacatatg	SLIC
SmaBamCas10	GAATTCACTGGCCGTCGTTTTACAggatcctcattccgattcttc	SLIC
BamSmaACYC	GGATCCTGTAAAACGACGGCCAGTGAATTctttaataaggagatataccatggctc	SLIC
SacHindCsm2	GCTCGACTGGGAAAACCCTGGCGAAGCTTctcacttgtctttcgggtcc	SLIC
HindSacACYC	AAGCTTCGCCAGGGTTTTCCCAGTCGAGctttaataaggagatataccatggctc	SLIC
XhoT7Cas10	CTTTGTTAGCAGCCGGATCTCTCGAGctcattccgattcttctttacg	SLIC
XhoForT7	ctcgagagatccggctgctaacaaag	SLIC
XhoForLac	ctcgagactagttccgtttaaaccc	SLIC
NdeVecRev	catatgtatatctccttcttaaagttaaac	SLIC
SmaBamCsm3	GAATTCACTGGCCGTCGTTTTACAggatcctcaaaccgctgcc	SLIC
XhoLacCsm5	GGGTTTAAACGGAACTAGTCTCGAGctcactctgcacgacggatg	SLIC
SacHind_ mcaCsm2	GCTCGACTGGGAAAACCCTGGCGAAGCTTctcattcggtttctttgctgg	SLIC
SacHindCsm5	GCTCGACTGGGAAAACCCTGGCGAAGCTTctcactctgcacgacggatg	SLIC

XhoLacCsm6	GGGTTTAAACGGAACTAGTCTCGAGctcaacccaatggtgccatg	SLIC
NcoInsFor	ccctctagaaataattttgtttaactttaagaaggag	SLIC
CDFInsRev	CTTTGTTAGCAGCCGGATCTCTCGAgttcaaatttcgcagcagcgg	SLIC
NcoVecRev	ctccttcttaaagttaaacaaaattatttctagaggg	SLIC
Csm3_D35A-fw	cgccgttgCtaaaccggtagttcgtgatc	Muta-genesis
Csm3_D35A-RP	ccggtttaGcaacggcgccaattgcagaa	Muta-genesis
Cas10_H18A_D19A-f	gcttactgGCcgCtattggaaagcctgttcagc	Muta-genesis
Cas10_H18A_D19A-RP	ttccaataGcgGCcagtaagcagcctataatcg	Muta-genesis
Cas10_D630A_D631A-fw	cggtggggCtgCtgtgtttgttgtgggggcat	Muta-genesis
Cas10_D630A_D631A-RP	caaacacaGcaGccccaccggaataaatgatg	Muta-genesis
ara-NdeI-R	ACGTCCATATGgtattcctcctgctagccc	Primer
ara-AvrII-F	GACTCCTAGgagctgcatgtgtcagagg	Primer
TetR-FOR	GATCGCATGcatctaattggactagtagcccgcctaatg	Primer
TetR-REV	CTTAGGATCCGTtcaggtcgaggtggcccggc	Primer
RSFara-FOR	caaataggcatgcagcgctcttc	Primer
RSFara-REV	cttaggatccCTTGTCGTCGGTTCAGGGCAG	Primer
lacZ-pRAT-fw	gtttttttgggctagcaggaggaatacCCATGGGCACCATGATTACGC	Primer
lacZ-pRAT-RP	agcagcggtttctttaccagactcgaGCTATGCGGCATCAGAGCAG	Primer
mtbRepeat RNA	uGUCGUCAGACCCAAAACCCCGAGAGGGGACGGAAAC	5' 6-FAM™
target RNA	AAcgacucuagaggauccccggguaccgagcucgaauucCAAAGGCA	RNA
anti-tag RNA	AGGUcgacucuagaggauccccggguaccgagcucgaauucGUUUCCGU	RNA

P1.A26 RNA	agggucguuguuaagaacgacguuguuagaaguugguaugguggaga	RNA
A1 RNA	aggguauuauuuguuuguuucuucuaaacuauaagcuaguucuggaga	RNA

Table S3 Sequences of synthetic genes

**Name**	**Sequence 5' → 3'**
mtb_cas6 (with additional 5' sequence)	catatgaccgagcatctgtcacgacttactctgacgctggaagtggacgcgccgctggaacgcgctcgggtc<br>gccaccttgggcccgcacctgcacggagtcctgatggagtccattcccgccgattacgttcaaaccctgcata<br>ccgtgccagtgaacccgtactcacagtacgccctggctcgcagcaccacaagcttagaatggaaaatctcg<br>accttgacgaacgaagcccggcaacagattgtgggcccgattaatgatgcggcgttcgcggggttcagact<br>gcgcgccagcggcatcgccacccaggtaaccagccgcagtttggaacaaaatcctctctcgcagttcgccc<br>gtatcttttatgctcgccccgaaacacggaaattccgtgtggaattcctgacacccaccgctttcaagcagagt<br>ggcgaatatgtgttttggccggacccgcgacttgtattccaatctctggctcaaaagtatggcgcgatcgtgga<br>cggggaagaaccggaccctggtctgatcgccgaatttgggcagagcgtgcgtttatcagctttccgtgttgcgt<br>ccgcaccctttgcagtgggtgctgcccgcgtgccaggcttcacaggcagcgctacgtttactgttcgcggtgtt<br>gatacgttcgcgagttacattgctgctctgctgtggtttggtgaatttagcgggtgtggtattaaagcctctatgggt<br>atgggcgcaatccgtgtgcagcccctggcaccgagggaaaaatgcgtaccaaaacctctcgag
mtb_cas10	ccatggctcatatgaacccccagttgatcgaagcgattataggctgcttactgcacgatattggaaagcctgtt<br>cagcgcgcggcactgggttatccgggtcgtcactccgcgatcggtcgtgcctttatgaaaaaagtatggctgc<br>gcgattcacgtaacccctctcagtttaccgatgaagttgatgaggctgacattggcgttagtgatcgtcgcattct<br>ggacgccattagctaccatcattcctcggcactgcggaccgcggccgagaatggtcgcttagccgcggacg<br>cgccagcttatattgcctataacatcgccgctggcacggaccgtcgcaaagccgacagcgacgatgggcat<br>ggtgccagcacctgggacccagataccccttttatactctatgttcaatcgcttcggtagtggaaccgctaacct<br>ggcatttgctccagaaatgctggacgaccgtaagcccatcaacatccctagcccgcgccgtatagaatttgat<br>aaagatcgttatgcggcgattgtgaataaactcaaagcgattctggtggatcttgagcgttcggatacctacctc<br>gcctcattattaaatgtgctggaggcgaccctgagcttcgtcccgagcagtacggatgccagcgaagtcgttg<br>atgttagtctttttgatcatcttaaactgaccggagcactgggcgcttgtatatggcattacctgcaagctactggt<br>caaagcgattttaaatcagccctctttgataaacaagacaccttttacaatgaaaaagcgtttttactgacgacc<br>ttcgacgtgtcaggcattcaagactttatttatacgattcactcaagcggcgctgcaaaaatgctgcgtgcacgt<br>agcttctatcttgagatgctgacggaacacttaatcgacgaattgctggcgcgtgtcggactgtcacgcgcga<br>atctcaactattccggcggcggtcatgcctatcttttgcttccgaacactgaaagcgcgcgtaagtcagttgaac<br>agtttgaacgtgaagcaaatgattggttacttgaaaatttcgccacccgtctgtttattgcgactggctcggtacct<br>ctggcggctaacgaccttatgcgtcgccccaacgaatcggcctctcaggcctctaatcgcgcactgcgctatt<br>caggcctgtatagagaactcagcgaacagctgtcagcaaagaaactggcccgctactctgcggaccagct<br>gcgcgagctgaacagtcgtgatcatgacggtcagaaaggagaccgtgaatgttctgtgtgccacactgtgaa<br>ccgaacggtgtcagcggatgacgaaccgaaatgtagcctttgccaggcgctgacggccgccagctcgcag<br>atacagtccgagagtcgccgctttttgctcatttcggacggagccactaaaggccttccgttaccgttcggcgct<br>acgctgactttctgttcgcgcgcagacgcagataaagcattgcaacaaccccagacgcgccgccggtatgc<br>taaaaacaagttctttgcgggcgaatgcttaggcaccgggctctgggtaggtgattatgttgcccagatggagt<br>tcggcgattacgtgaagcgggcttcgggtattgctcgcctgggcgtcctgcgtctggacgtcgataacctgggc<br>caggcgtttacacatggtttcatggagcagggaaatggcaagtttaataccatcagccgcaccgccgctttttc

	tcgcatgttgtcgctgttctttcgccagcacatcaactatgtgctggcacgtccgaaactacgcccgattaccgg<br>agacgatccggcccggccgcgtgaagcaaccatcatttattccggtggggatgatgtgtttgttgtgggggcat<br>gggatgatgttattgaatttggtatcgagctgcgcgaacgcttccatgaattcacgcaaggtaaacttaccgtat<br>ctgccgggatcggtatgtttccggacaagtatcctattagtgtgatggcccgcgaagtgggggacttagaaga<br>cgccgctaaatctctgcctggtaagaatggcgttgcattatttgatcgtgaattcactttcggttgggacgagctc<br>ctttctaaggtgattgaagaaaagtatcgtcatatcgcagattatttttccggcaacgaggaacgcggtatggc<br>gtttatttataaactgttagaattacttgcggagcgggatgatcgtattaccaaagcaaggtgggtgtactttctga<br>ctcgcatgcgcaatcctacgggcgatacggccccttccaacagttcgctaaccgtttacatcaatggtttcag<br>gacccgactgatgccaaacaactcaaaacggcactgcacctatatatttaccgcacacgtaaagaagaatc<br>ggaatgaggatccctcgag
mtb_csm2	ccatggctcatatgagtgtcattcaggacgattatgtgaaacaggccgaggtgattcgtgggctgccaaaaa<br>agaaaaatggattcgagctgaccaccacgcaattacgcgtgctcctgtccctgacggcccagctgtttgacg<br>aagcacaacagtcggccaatccgacgctgccgcgccagcttaaagaaaaagttcagtatctgcgtgtgcgc<br>ttcgtgtaccagtccggccgtgaagatgccgtgaaaaccttcgtacgcaatgcgaagctgttggaagctctag<br>aaggaattggcgactcgcgtgatggccttctacgtttttgccgatatatggaagcgctcgctgcatacaaaaag<br>tacctggacccgaaagacaagtgaggatccctcgag
mtb_csm3	ccatggctcatatgactacgagctacgcaaaaatcgaaattactggtacgctgactgtcctgaccgggcttca<br>gatcggcgctggcgatggattttctgcaattggcgccgttgataaaccggtagttcgtgatccgctgagtcggct<br>gcctatgatccccggtacgtctcttaaaggtaaagtccgcacgttattgtctcgccagtacggggcggacacc<br>gagacattctaccggaaaccaaacgaagatcatgcccatattcgacgtttattcggcgatacggaagaatat<br>atgaccggtcgccttgtgttcagagacaccaaactgaccaacaaagatgaccttgaggcgcgggggggcta<br>aaaccctgacggaagtaaaattcgaaaacgctattaaccgtgtgacggctaaagcgaacttacgccagatg<br>gagcgtgtgattccagggtctgaatttgccttttcgttggtctatgaagtcagttttggtaccccaggtgaagaac<br>agaaagcctctttaccttcttctgacgaaattatcgaagactttaatgccattgcccgcggcttgaaattattaga<br>attagactatttaggcggctctggcacccgcggctacggtcaggttaaattttcgaacctgaaagcacgagcc<br>gcggtaggtgccctggatggctcactcctggaaaagctcaaccacgaactggcagcggtttgaggatccctc<br>gag
mtb_csm4	ccatggctcatatgaactcacggctgtttcgttttgatttcgatcgtacgcacttcggcgatcacgggctggaaa<br>gctcaaccatcagttgtccggctgatacgctgtattcggcgctttgtgtcgaagccctgcgcatgggggccaa<br>cagctgctcggagagttggtagcctgtagtacactgcgcctgacggatctcctcccatacgttggacctgacta<br>cctggtccccaagccattgcattcagtccgcagtgacggctccagtatgcaaaaaaagctggcaaagaaga<br>tcggattcctgcctgctgcgcagctgggttcctttcttgacgggaccgcggacttgaaagaactggctgcgcgt<br>cagaccaaaattggagtccatgccgtttcggccaaagcggccatccataacggaaaaaaggatgcagatc<br>cgtatcgagtgggctatttccgtttcgagctagacgcagggctctggctgctagcaaccggtagtgagagcga<br>gttgggtttgctgacgcgccttctcaaaggtatctctgctctgggcggagaacgaacgtccggcttcggcgcctt<br>caatctcaccgagagtgaagctcctgctgccctcacaccgaccgtcgatgcagctagcttaatgacccttact<br>acctccctgcctactgatgacgaactcgaagccgcgttagccggcgcgacttatcgcttggttaaacgtagcg<br>ggtttgtagcttccagtacctatgccgatatgcctttgcgcaagcgtgatatctataaattcgctgctggatctgtgt<br>tttcccgcccgtttcagggtggtattctggatgtaagcttaggcggtaatcatccggtctattcgtatgctcgcccg<br>ctgttccttgcgctgccagaaagtgcagcatgaggatccctcgag
mtb_csm5	ccatggctcatatgaatacttacctgaagccattcgagctgacgctgcgttgtctgggccctgtcttcattggcag<br>cggcgagaaacggactagcaaagaataccatgttgaaggtgaccgcgtgtactttcctgatatggaactgct

	ctatgctgacattccagctcacaaaagaaaaagcttcgaagccttcgtaatgaacacggacggagcacaa<br>gcaacggcaccactgaaagagtgggtagagccaaatgcggtcaaattagatccggccaagcatcgtggct<br>acgaagttaaaattggcagcatcgaacctcgccgggcctctcgtggccgaggtggtcgtatgactcgtaaga<br>aactgactctaaacgaaattcacgccttcatcaaagacccgctgggtcgcccgtacgtcccgggttccacggt<br>aaaaggcatgttgcgctcaatctatctgcaaagtctggtacacaaacgcacagcgcaaccggtacgcgttcc<br>gggccaccagacgcgggaacaccgtcaatacggtgagcgcttcgagcgaaaggaactccgcaagagcg<br>ggcgccccaatacgcggccccaggatgctgtgaatgatctgttccaggcgattcgggttaccgattctccggc<br>cttacgtacaagcgacttgctgatttgtcagaaaatggacatgaacgttcatggcaaaccggacggcctgcc<br>gcttttccgcgaatgccttgcgcctggcactagcatctcacatcgtgtggtggttgacacctcccccacggcgc<br>gtggaggctggcgtgaaggcgaacgattcctggaaacgctcgcagaaacggctgccagcgtcaaccaag<br>ctcgttacgccgagtatcgcgcgatgtatccaggcgttaacgcgatcgtaggccctatcgtatacttaggaggt<br>ggggcaggctaccgcagcaagacttttgtgaccgaccaggatgacatggccaaagtcttagacgctcaattt<br>ggtaaagttgtgaaacacgtggacaaaacccgtgaattgcgtgtttccccgctcgttttgaagcgaaccaaaa<br>tcgataatatttgttatgaaatgggccaatgcgaactcagcatccgtcgtgcagagtgaggatccctcgag
mtb_csm6	ccatggctcatatgatcctgtttagcccgattgggaccgccgatccgatcactgcgctgggcgatggtcccatg<br>ctgcatattgtgagacattatcgtcctattgtggtcgtgctgtttctgagtgcagagatcgccgccttcgagaacg<br>cggatcgtcgctactccgcggctattacgcgtttagctccggaaaccgacgtgcgcattgtgacttatacgaat<br>ccaagcgttcaccggtttgacctgtttgttcctgtgttccgtaaccatttagtggaattaagcgcagaattccccga<br>ccgcacgatcctgttgaacaccagtagcggtacgcctgcgatgcaagcagcacttgtagccattaacgtattt<br>ggcatcccgcgtacaacggctgtacaagtgtcgaccccggcccgcgcgctgtctaaaccaggggatcgcg<br>aaagtcctgatgcttacgacctggaattaatgtgggatgcgaatgatgacaatcagccgggtgcccctaacc<br>gttgctttgaagccacatcggccgccctgggcgccctgctggaacgtgccaatctgaaacaactgattgttag<br>ctatgactattcggccgctgtgaccatcgctgcagatagccggctgccggaccaagtttcgaatctaattcgcg<br>gtgcaatgcatcgtagtcgtctcgaacatctggttgcccccaaattcttcaaagacaccgcattcacctatgac<br>ccggctaacaaagttgccgaatacatcagtgcacttgctttattggccaaacgcgaacaatgggcagaatttg<br>cgcgtagtgccaccccggcaattacaattgttcttcgcgccgcggtcgctaagcacctgccggaagatcgat<br>acctggatgatatgggtcgtgttgatcggcggaagctggagcgtgaaccagaaattcgctgcgcactaaagc<br>atccgcctaaaagtccgaatgcggaatggtatctgtacaccaaagattggttagccctcttgcgccagtttgcg<br>cctgatcgtgtgggtgcgctagaggttctgggccgtttcgagtcgcgtgtgcgtaataccgcggcgcatgaaat<br>tgtcagcatctcggaggatcgtatcaccaaagatggcggcctactgcccgaacagctgctgaagattttagcc<br>cgtgaaacgggcgcagatctaaccctgtacgatcgtctgaacgatgaaattatacgccagattgacatggca<br>ccattgggttgaggatccctcgag
mca_csm2	ccatggctcatatgagcgtgattcaggatgattatgtgaaacaggcggaacaggtgattcgcggcctgccga<br>aaaaaaacggcgattttgaactgaccaccacccagctgcgcgtgctgctgagcctgaccgcgcagctgtttg<br>atgaagcgcagctgagcagcgatcagaacctgagcccggcgctgcgcgataaagtgcagtatctgcgcgt<br>gcgctttgtgtatcaggcgggccgcgaaaaagcggtgcgcgtgtttgtggaacgcgcgggcctgctggatga<br>actggcgcagattggcgatagccgcgatcgcctgctgaaattttgccattatatggaagcgctggtggcgtata<br>aaaaatttcttgatccgaaagaaaccagcaaagaaaccgaatgaggatccctcgag
Tsu_csx1	ccatggatcgccatactcttatcacattcttaggcaaaggagctcgcgatcccaataccgggtatcgtagtacg<br>acgtaccgctttgcagatcgcgaccgttcgggtgcgtttttggactgcttcttgcacaacacctggctccagatc<br>gcttactgatcttgggaacccgctcttcccagtgggatctgttagttgagagtttagcagggtcgggggaggat<br>gaagaagctcgtttggcgttgatggaagccgtgagcgagggcgcagtgaccccacgcttgttagaacgtgtt

	cgccctttaatggaacgcgcggtcggcatcccattaaccccgtgcttgattccttttggggaagcccaagatgagcagcttgcaatcttagacactattgcaactcatgtcccgggtggcgaagtttcctttgacctgacgcatggtttccgccacatggggatgctggggttcctttctgcgttcatgttggagcgtattggacaccttcgtgttcgtggtctttggtatggcgctttagatatgtcacgcgataatatcacgccagttgtccgtctggatgggttatcagcggtccgtcattggattgaagcactttcccactttgaagcaactggcgattatggtgtgttcgcaagcctgttggtagcggatggcgtggcagaggataaagcccgttgcttagaggaagctgcatttcacgaacgcactttcaatttgttggatgccgttcgtaagttgcgtacgtttctgccggttcttgataccccattgggtggcgctagcggactttttcaggccgtgctgttacgccacttagaatggagtcgcgaatcttctctggacgcttatcagaagcgcttgtcacgcgcttacctgcacatgggagattttgtgcgcgccgttatctttggctgggaggctgttatcacacgtcactgtcttgcgaccggccgtgatccgcgtgacttcgcattgggtcgcaaggctgcggctgaagatttagacgcggacgttcgtaacggtattcgcgatcgcgattttgtacaagcctattggactattaagaacttgcgtaattcgctggcccacgggaacccaccaaaagataaggatcttctgcgcgtattgaaatccgaacaggatactcgtcgcttcattactgagtccttacaacgcctttagactgaggatcc
